# Spatiotemporal pattern of appraising social and emotional relevance: Evidence from event-related brain potentials

**DOI:** 10.3758/s13415-018-0629-x

**Published:** 2018-08-21

**Authors:** Annekathrin Schacht, Pascal Vrtička

**Affiliations:** 1 Affective Neuroscience and Psychophysiology Laboratory, Institute of Psychology, University of Goettingen, Gosslerstrasse 14, D-37073 Goettingen, Germany; 2 Leibniz ScienceCampus “Primate Cognition”, Kellnerweg 4, D-37077 Goettingen, Germany; 3 Department of Social Neuroscience, Max Planck Institute for Human Cognitive and Brain Sciences, PO BOX 500 355, D-04303 Leipzig, Germany

**Keywords:** Affective picture processing, Social content, Emotional valence, Appraisal, Relevance, Event-related brain potentials (ERPs)

## Abstract

Social information is particularly relevant for the human species because of its direct link to guiding physiological responses and behavior. Accordingly, extant functional magnetic resonance imaging (fMRI) data suggest that social content may form a unique stimulus dimension. It remains largely unknown, however, how neural activity underlying social (versus nonsocial) information processing temporally unfolds, and how such social information appraisal may interact with the processing of other stimulus characteristics, particularly emotional meaning. Here, we presented complex visual scenes differing in both social (vs. nonsocial) and emotional relevance (positive, negative, neutral) intermixed with scrambled versions of these pictures to *N* = 24 healthy young adults. Event-related brain potentials (ERPs) to intact pictures were examined for gaining insight to the dynamics of appraisal of both dimensions, implemented within the brain. Our main finding is an early interaction between social and emotional relevance due to enhanced amplitudes of early ERP components to emotionally positive and neutral pictures of social compared to nonsocial content, presumably reflecting rapid allocation of attention and counteracting an overall negativity bias. Importantly, our ERP data show high similarity with previously observed fMRI data using the same stimuli, and source estimations located the ERP effects in overlapping occipitotemporal brain areas. Our novel data suggest that relevance detection may occur already as early as around 100 ms after stimulus onset and may combine relevance checks not only examining intrinsic pleasantness/emotional valence but also social content as a unique, highly relevant stimulus dimension.

Humans are highly social beings (Aronson, 1980; Tomasello, 2014). Hence, social information is assumed to be of particular intrinsic relevance to humans due to its direct link to guiding physiological responses and behavior (Hariri, Tessitore, Mattay, Fera, & Weinberger, 2002; Keltner & Kring, 1998). A prominent evolutionary theory, the social brain hypothesis, even postulates that primates—including humans—have evolved unusually large brains for body size compared with all other vertebrates as a means to manage their unusually complex social systems (Dunbar, 1998, 2009). During the last two and a half decades, much research has thus been dedicated to better understand the functioning of the so-called social brain in a newly emerging field termed social cognitive affective neuroscience (Adolphs, 2003; Cacioppo & Berntson, 1992; Lieberman, 2007). Along these lines, social stimuli are argued to constitute the most emotionally evocative stimuli for humans, providing vital clues for survival throughout the life span by promoting both affiliative (e.g., attachment, reproduction) as well as protective (e.g., vigilance toward threatening encounters, protection of territory and significant others) behaviors (Insel, 2010; Norris, Chen, Zhu, Small, & Cacioppo, 2004; Porges, 2003). Accordingly, social interactions are thought to be motivated by emotions directing long-term social goals that are embedded in structures of social relationships, intentionality, and meaning. Conversely, in the nonsocial domain, emotions are likely to promote individual survival by maintaining immediate physiological and behavioral resources to biologically significant stimuli in terms of basic approach versus aversion responses (Britton et al., 2006; Insel, 2010; Porges, 2003).

Against this background, it is likely that the degree of social content of information may constitute a fundamental and distinct stimulus dimension, and that the human social brain may be highly sensitive to the mere presence of social information (Tso, Rutherford, Fang, Angstadt, & Taylor, 2018). A number of fMRI studies have therefore examined the potentially different neural substrates of social versus nonsocial information processing by comparing it to the neural processing of other stimulus dimensions, particularly emotional content in terms of a positive versus negative (versus neutral) hedonic valence dissociation (Britton et al., 2006; Frewen et al., 2010; Goossens et al., 2009; Hariri et al., 2002; Norris et al., 2004; Scharpf, Wendt, Lotze, & Hamm, 2010; Vrtička, Sander, & Vuilleumier, 2011, 2013). Several of these fMRI studies found brain areas showing preferential processing of social versus nonsocial information—including the occipital cortex/fusiform gyrus, amygdala, superior temporal sulcus, insula, and orbitofrontal cortex—and consequently indicated that neural processing of the social content dimension may occur in an additive or even an interactive manner with the emotional content dimension. Only one fMRI study (Vrtička et al., 2013), however, so far directly tested this assumption and found that neural processing of social and emotional content interacted distinctively in bilateral amygdala, right fusiform gyrus, right anterior superior temporal gyrus, and ventromedial prefrontal cortex. In all four brain areas, there was a fundamental social > nonsocial activation difference for emotional (positive and negative) images, with the same effect being present for emotionally neutral images. Furthermore, a social by emotional content interaction in brain activity arose (for positive and negative stimuli): activity in response to images of social content did not significantly differ between positive and negative valence, while activity for nonsocial images displayed a negative > positive valence effect. Described in other terms, there was a significantly larger social versus nonsocial activation difference for positive as compared with negative images. Importantly, this interaction was independent of low-level stimulus properties such as spatial frequency, contrast, and luminance, as well as arousal. Together, these findings by Vrtička et al. (2013) corroborate the notion that social content represents a fundamental and distinct stimulus dimension, and that information pertaining to the social versus nonsocial nature of stimuli is integrated with information regarding their emotional content.

In the present study, we aimed at further characterizing the interaction between social and emotional content during complex visual scene processing. More specifically, we focused on its underlying spatiotemporal pattern by means of event-related brain potentials (ERPs) while applying a very similar experimental design as implemented by Vrtička et al. (2013) using fMRI. Because of their excellent temporal resolution, ERPs provide a powerful tool to investigate the processing specificities triggered by different types of relevance over time. The most prominent ERP components sensitive to emotional relevance are the early posterior negativity (EPN) and the late positivity complex (LPC), with the latter often likewise termed as late positive potential (LPP; e.g., Schupp et al., 2004). The EPN, which occurs as a relative negativity over posterior electrode sites starting around 150–200 ms after stimulus onset, has been proposed to reflect enhanced sensory encoding resulting from involuntary capture of attention by various stimuli of emotional content, (e.g., Bayer & Schacht, 2014; Junghoefer et al., 2001; Schacht & Sommer, 2009a, 2009b; Schupp et al. 2007). The LPC/LPP has been linked to higher-order stages of stimulus evaluation, developing around 300 ms and typically lasting for several hundred milliseconds (e.g., Schacht & Sommer, 2009a). Complementing these findings, there is growing evidence indicating prioritized processing of emotionally relevance stimuli to start already at early sensory stages. Several studies demonstrated the amplitudes of the visual C1 (peaking around 80 ms) and P1 (peaking around 100 ms) components to be enhanced for emotional compared with neutral stimuli (Batty & Taylor, 2003; Brosch et al., 2008; Holmes et al., 2009; Ortigue et al., 2004; Pourtoiset al., 2004; Rellecke, Sommer, & Schacht, 2012; Rossi et al., 2017; Stolarova, Keil, & Moratti, 2006).

In contrast to the well-documented ERP modulations by emotional relevance, effects of other sources of relevance, including social relevance, and their integration with emotional aspects have been largely neglected to far. Only one previous EEG study (Okruszek et al., 2016) explicitly aimed at differentiating social from nonsocial content in addition to testing for emotional content effects using complex visual scenes, although this study only comprised stimuli with a negative versus neutral valence. The authors reported early effects of social content at the P1 (social > nonsocial) and at the EPN (nonsocial > social) component. Later stages of processing were only impacted by emotional content, as indicated by larger P3 and LPP amplitudes for negative than for neutral picture content. Interactions between social and emotional content were restricted to the N2 component, with extenuated amplitudes for negative pictures with social content compared with all other picture conditions. Although these findings provide first insight into the temporal dynamics of the processing of social and emotional content, they lack information on the neural processing of positive valence and are further inconclusive due to highly unconventional choice of electrodes and quantification of ERP amplitudes. Together, it remains an open question in which temporal sequence different stimulus dimensions are processed by the human brain, and what significance such processing sequence may have for physiological responses and behavior.

One theoretical framework that is devoted to addressing this question is the component process model of emotion developed by Scherer and colleagues (see, e.g., Sander, Grandjean, & Scherer, 2005; Scherer, 2009), the latter model being situated within the larger realm of appraisal theories of emotion. The component process model of emotion proposes a sequence of appraisal checks that coordinate a range of responses to a particular event. Within this approach, the detection of relevance is considered to be “a first selective filter through which a stimulus or event needs to pass to merit further processing” (Scherer, 2009, p. 3463), and to comprise information evaluation in terms of novelty (i.e., suddenness, familiarity, and/or predictability), intrinsic pleasantness (i.e., negative vs. positive [vs. neutral] valence), and goal / need relevance (i.e., whether the assessed information accords to or obstructs the current goals and needs of the organism).

First evidence that such temporal sequence of stimulus appraisal—particularly related to relevance detection—is implemented at the brain level was provided by an ERP study, which investigated the neural unfolding of effects of novelty and intrinsic pleasantness by means of negative, positive, and neutral images during an oddball task (van Peer, Grandjean, & Scherer, 2014). The authors reported a novelty effect arising first in ERPs between 200 and 300 ms, followed by an intrinsic pleasantness effect between 300 and 400 ms, and finally a novelty by intrinsic pleasantness interaction between 700 and 800 ms. The temporal dynamics of these effects indicate that the processing of intrinsic pleasantness, that is, the emotional content in terms of positive versus negative (versus neutral) valence, is appraised relatively early during the temporal sequence and thus constitutes one of the first relevance checks, although not the very first one.

Concerning the processing of social content, no study has yet assessed its temporal unfolding over time in the context of relevance detection and the appraisal theory of emotion. As already mentioned above, however, Okruszek et al. (2016) provided preliminary evidence for early effects of social content at the P1 (social > nonsocial) and at the EPN (nonsocial > social) component, regardless of stimulus valence. Such early social relevance effect in terms of a social versus nonsocial activation difference during complex visual scene processing is corroborated by a recent study using negative versus neutral written sentences as stimuli, and manipulating the social content dimension by means of social closeness (i.e., whether the sentences referred to participants’ significant others or to unknown agents) (Bayer et al., 2017). The authors also report early social content effects in ERPs in the P1 component (from 73 to 120 ms), irrespective of the sentences’ emotional valence. Furthermore, the authors report an effect of emotional content at a later stage in the EPN component (from around 200 ms on), and an interaction between social and emotional content in terms of the EPN having a longer duration when emotional words were presented in highly relevant social contexts, that is, referring to the participants’ boyfriend or best friend. Despite differences in the way the social content dimension was characterized, these data together indicate that a relevance check pertaining to social content may also occur early during stimulus appraisal—already at the P1 component—and that an interactive processing of social and emotional content may follow at the EPN component and/or later on.

By applying a very similar experimental design as implemented by Vrtička et al. (2013) using fMRI, and according to theoretical considerations and available data on the temporal dynamics of social and emotional content processing using EEG outlined above, we predicted that social content might constitute a distinct stimulus dimension to be appraised in a relevance check separate from hedonic pleasantness. Specifically, we anticipated effects of social content to occur early during stimulus processing, presumably modulating already the P1 component of ERPs. We also predicted modulation of ERP responses by emotional content, reflecting another relevance check. Although there are reports of emotional content effects occurring as early as around 100 ms after stimulus onset, the two previous ERP studies directly manipulating social and emotional content demonstrated emotion effects at subsequent ERP components, namely the N2, EPN, and P3, respectively. We therefore assumed a temporal sequence in the order of social content followed by emotional content appraisal. Finally, in line with previous fMRI data (Vrtička et al., 2013), we expected to find a robust interaction between social and emotional content according to the pattern described above that was present in bilateral amygdala, right fusiform gyrus, right anterior superior temporal gyrus, and ventromedial prefrontal cortex. While scalp-surface ERPs cannot capture neural activity in the amygdala, we anticipated to observe a social by emotional content interaction in cortical areas like the fusiform gyrus and/or anterior superior temporal gyrus.

Along the above lines, and to keep as closely as possible with the experimental design of the fMRI study by Vrtička et al. (2013), we first analyzed the ERP data for social image content (2; social, nonsocial) and emotional image content (2; positive and negative) separately from data for neutral image content (2; social and nonsocial). Because other ERP studies usually also include neutral stimuli as a direct comparison condition, we subsequently computed a second analysis with a full 2 (social content) × 3 (emotional content) factorial design. The image rating as well as reaction time data, however, is presented with a full 2 × 3 factorial design from the start.

## Method and materials

### Participants

The experiment was conducted with 24 participants ranging in age between 20 and 33 years of age (*M* = 25.71 years, *SD* = 3.42). Only female participants were accepted for participation, as emotion and social content effects may differ between sexes, with females tending to show stronger effects (Bennett, Farrington, & Huesmann, 2005; Federmeier, Kirson, Moreno, & Kutas, 2001). All participants had normal or corrected-to-normal vision and, according to the Edinburgh’s Handedness Inventory (Oldfield, 1971), were right-handed. Participation was voluntary, and the study was approved by the ethics committee of the Institute of Psychology, University of Göttingen. Participants gave written informed consent prior the study and were reimbursed for participation.

### Materials

Out of a set of 360 previously validated colored images depicting complex visual scenes (Vrtička et al., 2011, 2013), 120 pictures were chosen for the current study. All images were collected from the International Affective Picture System (IAPS) or from free sources on the Internet. They varied in their social (social, nonsocial) and emotional content (positive, negative, neutral) resulting in six experimental conditions (with 20 images per condition). Positive social images included scenes that depicted parents interacting with their children, friends having a good time together, or happy moments in the context of romantic relationships. Social negative images comprised scenes where people were in distress due to a loss, interpersonal violence, sickness, or environmental circumstances. Nonsocial positive images illustrated animals (mainly cute animal babies), holiday scenes, and nice food. Nonsocial negative images also comprised animals (mainly scary or dead), natural disasters or accident scenes, or body parts with injuries. Finally, neutral social images showed portraits of people with a neutral facial expression or working individuals, whereas neutral nonsocial images mainly depicted objects like shoes, a book, or a chair.

**Fig. 1:**
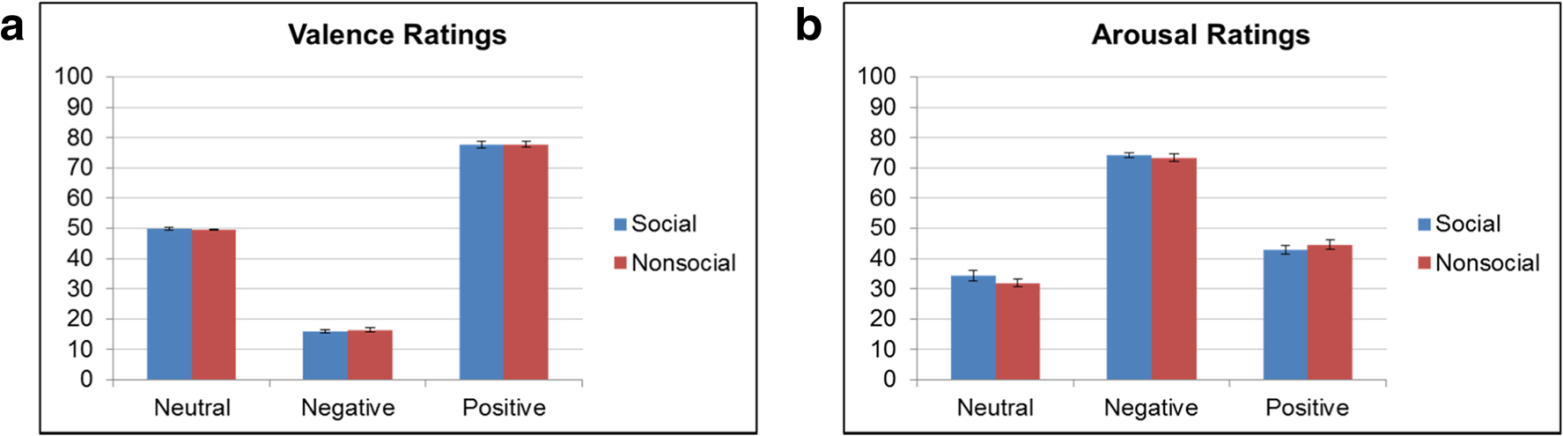
Valence and arousal image ratings. **a** Valence ratings (on a scale from 1 to 100; *y*-axis) for all six image categories. **b** Arousal ratings (on a scale from 1 to 100; *y*-axis) for all six image categories. Error bars represent 1 *SEM*

All 120 images were adjusted on several low-level image properties, and adjustment was verified by separate social content (2; social, nonsocial) by emotional content (3; negative, positive, neutral) repeated-measures analyses of variance (rmANOVAs). For luminance and contrast, one value was derived per image and entered in the 2 × 3 rmANOVA, with no significant main effects or interactions detected for luminance, *F*s(1, 19) < .635, *p*s > .54, or contrast, *F*s(1, 19) < .18, *p*s > .84. For spatial frequency, nine wavelet (Haar) coefficients reflecting increasing levels of spatial frequencies were derived for each image. We then tested the first three (i.e., highest) and last three (i.e., lowest) spatial frequencies separately with a 2 × 3 rmANOVA each. This procedure did not reveal any significant main effects or interactions, *F*s(1, 19) < 2.71, *p*s > .08. Hence, there were two frequencies (lowest of the high and highest of the low frequencies) where the *p* value of the main effect of emotional content was *p* < .10. For these two cases, we additionally checked post hoc effects, but these were not significant, *p*s > .07, either. This means that none of the six experimental conditions significantly differed from each other, even in those two frequency bands. We are therefore confident that any observed effects in the ERP signal cannot easily be explained by low-level image properties like luminance, contrast, and/or spatial frequency. Furthermore, emotional valence and arousal of images was controlled via preexperimental ratings for positive and negative social and nonsocial images (Vrtička et al., 2011, 2013), while the ratings for the neutral control condition images (neutral social, neutral nonsocial) were directly taken from the IAPS database, and these values were again entered in a 2 (social content) × 3 (emotional content) rmANOVA each. These analyses revealed no significant main effect of social content, *F*s(1, 19) < .282, *p*s > .602, and no social content x emotional content interaction, *F*s(1, 19) < 1.08, *p*s > .348, but a main effect of emotional content, *F*s(1, 19) > 532, *p*s < .001. This main effect of emotional content came about because there was a significant positive > neutral > negative difference in terms of valence ratings, and a significant negative > positive > neutral difference in terms of arousal ratings (all post hoc tests *p*s < .001; see Fig. 1a–b).

For the present experiment, scrambled versions of the 120 original images were created using Adobe Photoshop (Version 11; “Filter / Telegraphics / Scramble” command), thereby generating another set of 120 images consisting of 3,072 randomly distributed small squares each.

### Procedure

Before the start of the experiment, participants signed informed consent and provided demographic information. Stimuli were presented at the center of a computer screen (gray background), positioned at a distance of 90 cm from the participant. Stimuli presentation was controlled by Presentation® software.

The main experiment consisted of four blocks. Within each block, all 240 images—120 intact (targets) and 120 scrambled (distractors)—were presented in randomized order. The participants’ task was to indicate by button press whether the presented image was intact or scrambled. Response-by-button assignments were counterbalanced across participants. Each trial started with the presentation of a fixation cross for 2,500 ms, followed by the picture stimulus shown for 150 ms. After a blank of 850 ms duration, a question mark was presented for maximum 3,000 ms, indicating the time period for responses. Feedback (“too fast,” “too slow”) was provided in case of responses outside of this interval. With the button press, the next trial was initialized. Breaks were included after every 120 trials. In order to familiarize participants with the timing of stimulus presentation and procedure of the task, there were 10 practice trials (half distractors) prior to the experiment.

Picture stimuli consisted of 512 × 384 pixel (14 × 10.5 cm), corresponding to a visual angle of 8.8° × 6.7°. Fixation crosses, feedback stimuli, and question marks were presented in white color.

### Electrophysiological recordings and preprocessing

The electroencephalogram (EEG) was recorded from 64 electrodes placed in an electrode cap (Easy-Cap, Biosemi, Amsterdam, The Netherlands) according to the extended 10–20 system (Pivik et al., 1993). The common mode sense (CMS) electrode and the driven right leg (DRL) passive electrode were used as reference and ground electrodes (cf. www.biosemi.com/faq/cms&drl.htm). Six external electrodes were placed laterally and inferior to the eyes to record blinks and eye movements, and on the left and right mastoids. Signals were recorded at a sampling rate of 512 Hz and a bandwidth of 104 Hz, and off-line filtered with a low cutoff (0.03183099 Hz, time constant 5 s, 12 dB/oct), a high cutoff (40 Hz, 48 dB/oct), and a notch filter (50 Hz). Data were processed with BrainVision Analyzer (Brain Products GmbH, Munich, Germany). Data were average-referenced and corrected for blinks and eye movements using Surrogate Multiple Source Eye Correction (MSEC; Ille, Berg, & Scherg, 2002) as implemented in BESA (Brain Electric Source Analysis, MEGIS Software GmbH, Gräfeling, Germany). The continuous EEG signal was segmented into epochs of 1,100 ms, starting 100 ms before stimulus onset, and referred to a 100 ms prestimulus baseline. After rejecting epochs containing artifacts (criteria: voltage steps larger than 50 μV, 200 μV/200-ms intervals difference of values, amplitudes exceeding −150 μV/150 μV, and activity smaller than 0.5 μV), ERP segments were averaged per participant and experimental condition.

### Data analyses

Reaction times (RTs) were analyzed with a full-factorial repeated-measures analysis of variance (rmANOVA), including the factors social content (2; social, nonsocial) and emotional content (3; positive, negative, neutral). Accuracy was calculated as an average over all conditions and participants.

For EEG data analysis, data from practice trials, the first trial of each block, trials with erroneous or missing responses, and distractor trials (trials containing scrambled pictures) were discarded. In order to allow direct comparisons of results between the present ERP and the previous fMRI study (Vrtička et al., 2013), analyses were in a first step conducted for testing the social by emotional content interactions on positive/negative pictures and neutral pictures separately. An additional second analysis step was then computed, including all experimental conditions into one full factorial 2 (social content) × 3 (emotional content) rmANOVA.

**Fig. 2:**
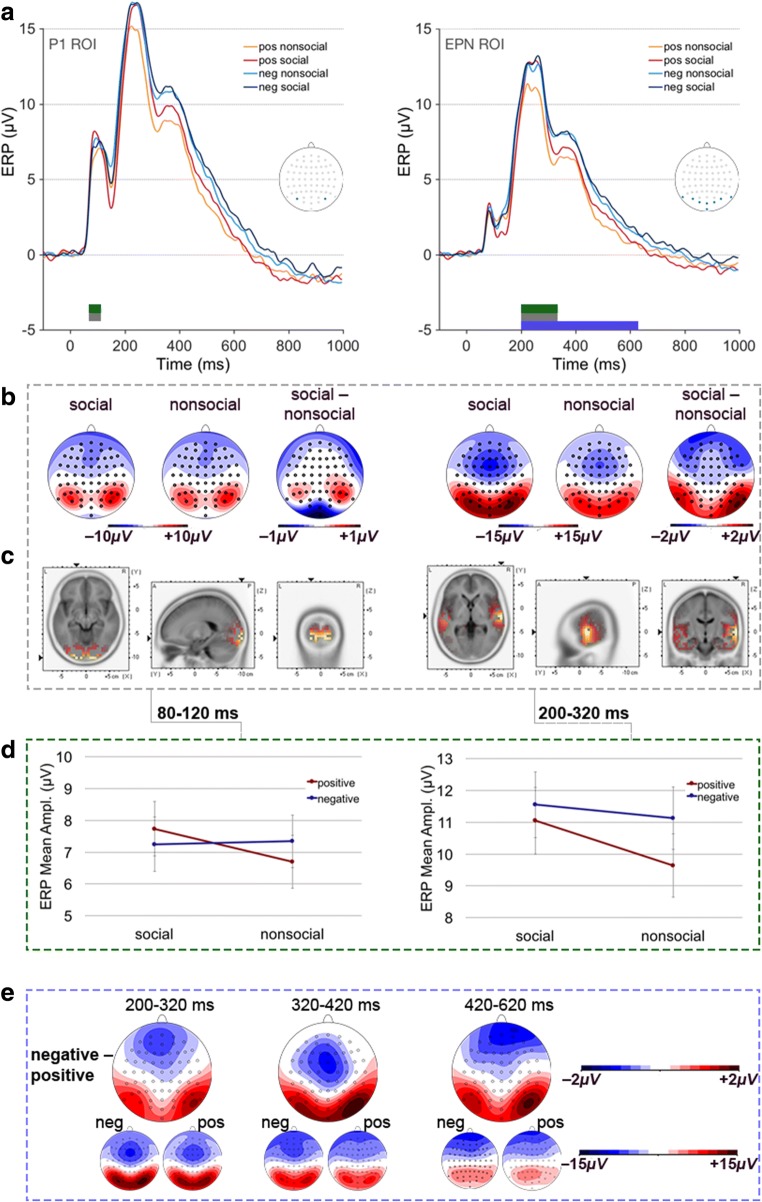
**a** Grand average ERPs, contrasted for social, nonsocial × positive (pos), negative (neg) picture content, time-locked to stimulus onsets at selected ROI electrodes. The colored bars above the *x*-axis mark the significant main effects of the factors social content (gray), emotional content (blue), and their interaction (green). Inserted heads highlight selected ROI electrodes. **b** Scalp distribution of ERP effects within the P1 (left) and EPN (right) time windows, and **c** respective source localizations of the social > nonsocial ERP differences within these intervals. **d** ERP mean amplitudes (with *SEM*s) for the social by emotional content interaction within the P1 (left panel) and the EPN (right panel) time windows. **e** Scalp distribution of the ERPs to positive and negative content (small maps) and of ERP difference waves between negative and positive content (large maps) within the three time windows of significant emotion effects. (Color figure online)

ERP data was analyzed as follows: Based on previous research and visual data inspection, time windows for ERP components of interest were chosen as follows: (a) P100 between 80 and 120 ms, (b) EPN between 200 and 320 ms, (c) P300 between 320 and 420 ms, and (d) LPC between 420 and 620 ms. The P100 component was quantified by mean amplitudes at PO7 and PO8 electrodes, where the component showed its maximal positivity. EPN amplitudes were averaged across electrodes P9, PO7, O1, Iz, Oz, O2, PO8, P10, covering typically involved posterior electrode sites (hereafter named posterior ROI). P300 and LPC mean amplitudes were first quantified at a cluster of parietal electrodes, including P3, P1, Pz, P2, P4, PO3, POz, and PO4 (parietal ROI). However, as becomes visible in Fig. 2e, the distribution of ERP components within the two latter time windows was shifted toward posterior sites, being highly similar to the preceding EPN time window. We therefore applied the same posterior ROI also to the ERP analyses during the P3 and LPC time intervals.

Mean amplitudes were analyzed with rmANOVAs, including the factors social content (2; social, nonsocial) and emotional content (2; positive, negative—and subsequently 3; positive, negative, neutral). In case of significant main effects or interactions between the experimental factors included, follow-up analyses were conducted with Bonferroni-corrected pair-wise comparisons.

In order to estimate the neural generators underlying the dominant voltage topographies identified at the scalp level, sLORETA (Pascual-Marqui, 2002) was used (sLORETA is a distributed linear inverse solution based on the neurophysiological assumption of coherent coactivation of neighboring cortical areas, that are known to have highly synchronized activity; Dasilva, 1991). Accordingly, it estimates multiple simultaneously active sources without any a priori assumption on the number and position of the underlying dipoles; sLORETA solutions are computed within a three-shell spherical head model coregistered to the MNI152 template (Mazziotta et al., 2001). It estimates the three-dimensional intracerebral current density distribution in 6,239 voxels of 5 mm spatial resolution. We performed comparisons on log-transformed data using paired-samples *t* tests in the time windows corresponding to relevant ERP effects. Only one single *t* test per voxel was performed per time window. Statistical analyses were based on a stringent nonparametric randomization (5,000 iterations), providing corrected *p* values. Given the low resolution of sLORETA, only brain areas showing a minimum of *k* > 20 significant voxels will be reported, together with coordinates referring to maximum activations within brain areas.

## Results

### Behavioral performance—Reaction times and accuracy

Reaction times (minimum, maximum, mean, and standard deviation) for all six intact picture experimental conditions are summarized in Table 1. A full factorial rmANOVA with the factors social (2) and emotional content (3) for intact images did not reveal any significant main effects or interactions (all *p*s > .30). Mean accuracy (percentage correct responses to the task—intact vs. scrambled decision) was very high at a value of 99.42 ± 1.06%.

**Table 1:** Reaction times for each intact picture experimental target condition

	Minimum	Maximum	Mean	*SD*
Positive nonsocial	222.82	583.3	379.70	111.58
Positive social	231.93	592.02	379.50	119.74
Neutral nonsocial	232.46	561.36	378.98	103.87
Neutral social	214.56	587.98	382.03	116.52
Negative nonsocial	205.77	612.86	372.70	114.25
Negative social	219.3	606.47	377.25	114.64

### ERP modulations by social and emotional content—Analysis conform with fMRI data

In order to allow direct comparison of results between the present ERP and the previous fMRI study (Vrtička et al., 2013), analyses were in a first step conducted for testing the social by emotional content interaction on positive/negative pictures and neutral pictures separately.

#### ERP effects of social content

Averaged ERPs contrasted for all emotional conditions (social positive, social negative, nonsocial positive, and nonsocial negative) are depicted in Fig. 2a. During the P1 time window (80 to 120 ms), the rmANOVA on mean amplitudes—quantified at electrode sites PO7 and PO8—revealed a significant main effect of social content, *F*(1, 23) = 7.522, *p* = .012, η_p_^2^ = .246, with augmented amplitudes for pictures of social content compared with pictures of nonsocial content, mean difference = 0.475, 95% CI [0.117, 0.833] (see Fig. 2d, left panel).

A similar pattern occurred during the EPN interval (200 to 320 ms)—quantified at posterior electrode sites—by means of a significant main effect of social content, *F*(1, 23) = 14.731, *p* = .001, η_p_^2^ = .390, which was again driven by larger posterior positivities of pictures of social in comparison with nonsocial content, mean difference = 0.92, 95% CI [0.424, 1.416], (see Fig. 2d, right panel). During the P3 (320 to 420 ms) and LPC (420 and 620 ms) time windows, there were no significant main effects of social content on posterior ERP activity, all *Fs* < 2.0, *p*s > .1.

As is visible in Fig. 2b (left panel), the scalp distribution of the early ERP modulation by social content resembled a typical P1 component with bilateral local maxima at occipital electrode sites, whereas during the subsequent time interval (EPN), a posterior positivity—instead of the expected enlarged negativity—occurred, extending to more temporal areas and accompanied by a stronger and more widely distributed frontal negativity (see Fig. 2b, right panel).

Source estimations for ERP activity in the two relevant time intervals confirmed this impression: While the early P1 modulation was mainly generated in occipital brain areas, sources of the subsequent ERP effect were located in more temporal brain areas (see Table 2 and Fig. 2c).

**Table 2:** Results of source analyses of the ERP effect of social content

	BA	Hem	Cluster size	*T* value (max)	Tal coord (max) *x, y, z*			BA (max)
SOCIAL > NONSOCIAL, between 80 and 120 ms								
Cuneus	17/18/19	L R	99 106	14.07 14.23	−10 10	−97 −97	1 1	17
Lingual gyrus	17/18/19	L R	72 77	14.63 14.18	−20 15	−78 −97	−5 −4	18
Middle occipital gyrus	18/19	L R	63 61	13.92 13.38	−20 20	−97 −97	5 5	18
Fusiform gyrus	18/19/37	L R	32 29	12.93 12.23	−25 20	−93 −93	−12 −12	18
Middle temporal gyrus	19/37/39	L R	13 26	5.84 6.90	−40 35	−82 −81	18 23	19
Precuneus	19/31	L R	12 34	5.57 6.99	−25 20	−72 −72	17 17	31
SOCIAL > NONSOCIAL, between 200 and 320 ms								
Superior temporal gyrus	22/41/42	L R	77 96	5.34 6.45	−54 64	−29 −14	6 5	22 22
Middle temporal gyrus	21/22	L R	70 90	5.32 6.54	−54 64	−29 −15	1 1	21 21
Inferior temporal gyrus	20/21/37	L R	54 43	4.96 5.75	−53 64	−30 −20	−15 −16	20 20
Fusiform gyrus	20/37	L R	49 20	4.73 4.82	−54 59	−35 −16	−19 −24	20 20

*Note.* The list was limited to brain regions showing *k* > 20 significant voxels in order to account for the low resolution of the sLoreta approach. BA = Brodmann area; Hem = hemisphere, Tal = Talairach

#### ERP effects of emotional content

P1 mean amplitudes were not affected by emotional content, *F*(1, 23) < 1 (see Fig. 2d, left panel). During the EPN time window (200 to 320 ms) within the posterior ROI, however, a main effect of emotional content occurred, *F*(1, 23) = 49.128, *p* = .0001, η_p_^2^ = .681, reflecting increased posterior positivities elicited by pictures of negative valence as compared with pictures of positive valence, mean difference = 0.999, 95% CI [0.704, 1.294] (see Fig. 2d, right panel). As is obvious from Fig. 2e, this posterior positivity sustained over the subsequent time intervals (i.e., between 320 and 420 ms and between 420 and 620 ms). The rmANOVAs revealed main effects of emotional content in both of these later time intervals, *F*(1, 23) = 31.118, *p* < .001, η_p_^2^ = .575, and, *F*(1, 23) = 36.731, *p* < .001, η_p_^2^ = .611, reflecting enhanced posterior positivities to negative pictures compared to positive pictures, mean difference = 1.304, 95% CI [0.821, 1.788], and mean difference = 1.176, 95% CI [0.771, 1.581] (not shown).

#### Social × emotional content interactions

During the P1 time window (80 to 120 ms), the rmANOVA revealed a significant interaction between social and emotional content, *F*(1, 23) = 9.910, *p* = .005, η_p_^2^ = .301 (see Fig. 2d, left panel). This interaction was driven by (a) pictures of positive valence showing a significant social > nonsocial difference in amplitudes, mean difference = 1.043, *p* = .001, 95% CI [0.495, 1.591], whereas no such effect was present for pictures of negative valence, mean difference = .095, *p* = .692, 95% CI [−.391, .578]; and (b) pictures with nonsocial content showing a significant negative > positive difference in amplitudes, mean difference = .647, *p* = .001, 95% CI [0.212, 1.082], whereas no such significant effect was present for pictures with social content, mean difference = .489, *p* = .075, 95% CI [−.053, 1.032].

During the EPN time window (200 to 320 ms) within the posterior ROI, we again observed a significant interaction between social and emotional content, *F*(1, 23) = 5.256, *p* = .031, η_p_^2^ = .186 (see Fig. 2d, right panel). Here, the interaction emerged because (a) pictures of positive valence showed a significant social > nonsocial difference in amplitudes, mean difference = 1.421, *p* = .001, 95% CI [0.652, 2.19], whereas no such effect was present for pictures of negative valence, mean difference = .42, *p* = .132, 95% CI [−0.136, 0.976]; and (b) because there was a smaller significant negative > positive effect for pictures of social content, mean difference = 0.499, *p* = .043, 95% CI [0.017, 0.981], as compared with nonsocial content, mean difference = 1.5, *p* < .001, 95% CI [0.908, 2.092].

The emotional × social content interaction did not reach significance during the P300 (320 to 420 ms) and LPC (420 and 620 ms) time intervals, all *F*s < 2.0, *p*s > .1.

#### ERP components to social content in neutral pictures

In order to test potential influences of social content on the processing of emotionally neutral pictures, mean amplitudes were quantified at ROI electrodes within time intervals indicated by significant main effects of social content in the analyses on ERPs described above.

**Fig. 3:**
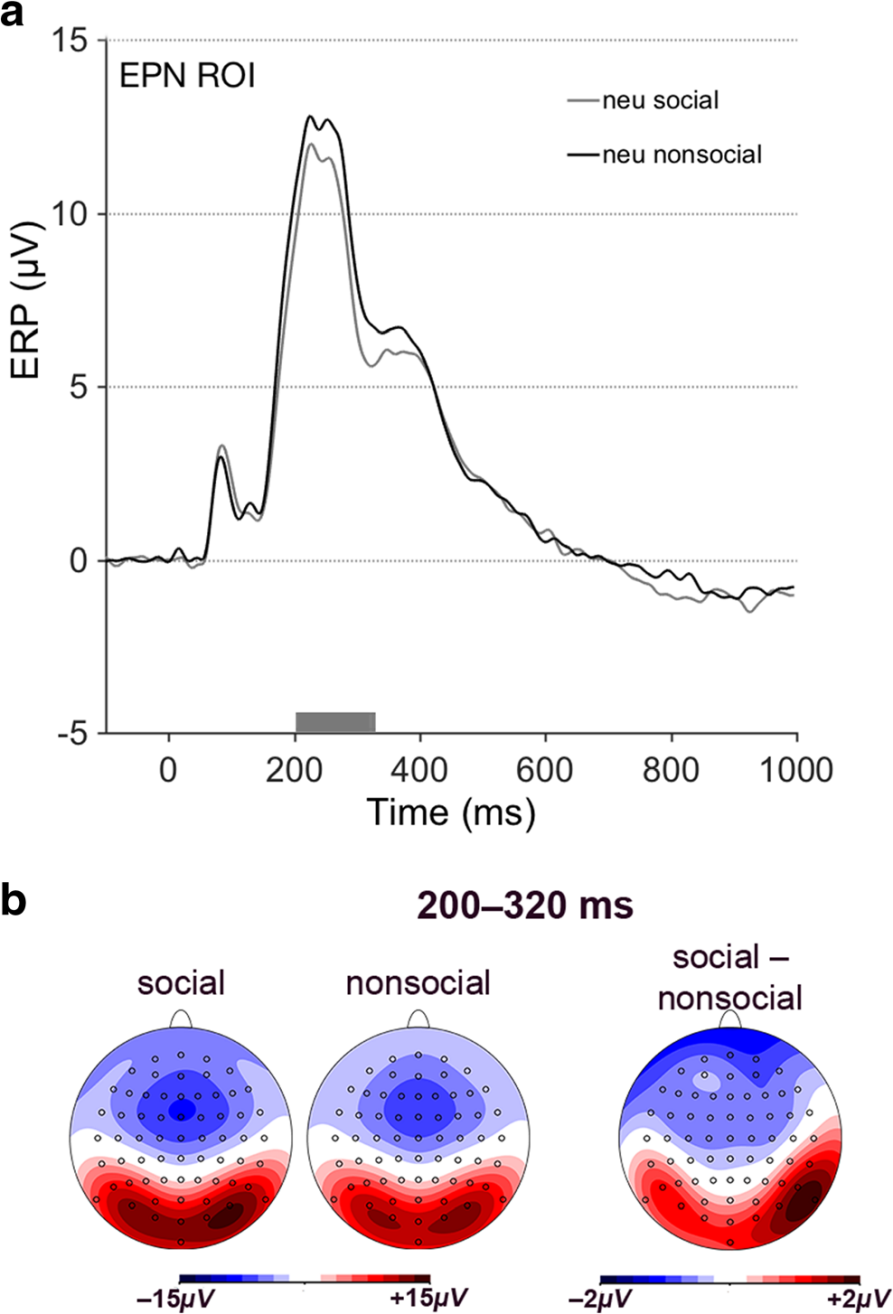
ERP effects of social relevance during neutral picture processing. **a** Grand average ERPs, contrasted for social and nonsocial content. The gray bar above the x-axis marks the significant main effect of the factor social content. **b** Scalp distributions of grand average ERPs and their difference between 200 and 320 ms; neu= neutral. (Color figure online)

The P1 component (between 80 and 120 ms) elicited by neutral pictures was unaffected by social content, *F*(1, 23) = .399, *p* = .534, η_p_^2^ = .017. Between 200 and 320 ms, pictures of social content elicited significantly larger posterior positivities than did pictures of nonsocial content, *F*(1, 23) = 21.648, *p* < .001, η_p_^2^ = .485, mean difference = 1.184, 95% CI [.658, 1.711], with highly similar scalp distribution as emotional pictures of social content (see Fig. 3). As for the P1 component, no significant social versus nonsocial effect for neutral pictures was present between 320 and 420 ms (P3 component), although there was a trend toward significance, *F*(1, 23) = 3.907, *p* = .060, η_p_^2^ = .145, and no significant effect was present between 420 and 620 ms (LPC component), *F*(1, 23) = 0.037, *p* = .849, η_p_^2^ = .002.

### ERP modulation by social and emotional content—Full factorial analysis

After a first analysis conducted for testing the social × emotional content interaction on positive/negative pictures and neutral pictures separately in order to allow direct comparisons of results between the present ERP and the previous fMRI study (Vrtička et al., 2013), an additional second analysis step was added by combining all experimental conditions into one full factorial 2 (social content) × 3 (emotional content) rmANOVA.

#### ERP effects of social content

**Fig. 4:**
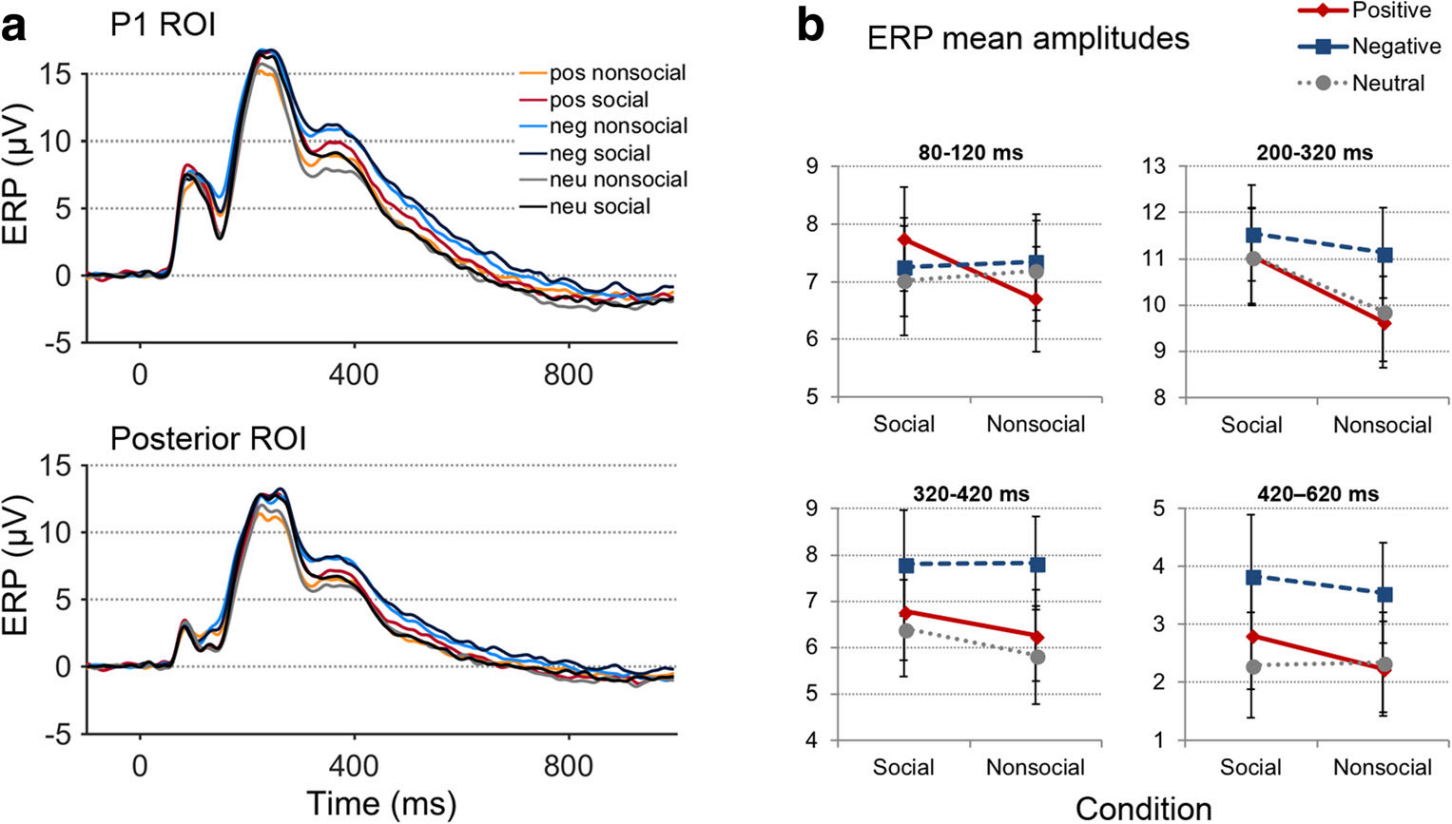
**a** Grand average ERPs, contrasted for all six experimental conditions, synchronized to stimulus onsets, at the P1 (upper panel) and posterior ROI (lower panel). **b** ERP mean amplitudes (with *SEM*s) during the four time windows of interest; pos= positive, neg= negative, neu= neutral. (Color figure online)

Averaged ERPs during the four assessed time windows comprising all three emotional content conditions only revealed a significant main effect of social content during the EPN time window (200–320 ms), quantified at posterior electrode sites, *F*(1, 23) = 24.787, *p* < .001, η_p_^2^ = .519 (see Fig. 4b). The respective relevant post hoc tests are summarized in Table 3.

**Table 3:** Summary of relevant post hoc *t* tests for the main effect of social content and emotional content, as well as the social × emotional content interaction, using the full-factorial (2 × 3) analysis

Post hoc tests (Bonf. corrected)	Mean diff.	*p* value	95% CI Low	95% CI High
P100 (80–120 ms)				
Social > nonsocial for negative	0.094	.692	−0.391	0.578
Social > nonsocial for positive	1.043	.001 ***	0.495	1.591
Social > nonsocial for neutral	0.177	.534	−0.403	0.758
Negative > positive for social	0.489	.225	−0.188	1.167
Negative > neutral for social	0.235	1	−0.483	0.954
Positive > neutral for social	0.725	0.058	−0.02	1.469
Negative > positive for nonsocial	0.647	.016 *	0.104	1.19
Negative > neutral for nonsocial	0.152	1	−0.326	0.629
Positive > neutral for nonsocial	0.496	.06	−0.016	1.008
EPN (200–320 ms)				
Social > Nonsocial	1.008	<.001***	0.589	1.427
Negative > Positive	0.999	<.001***	0.631	1.368
Negative > Neutral	0.878	.001***	0.32	1.436
Positive > Neutral	0.122	1	−0.328	0.571
Social > Nonsocial for negative	0.42	.132	−0.136	0.976
Social > Nonsocial for positive	1.421	.001***	0.652	2.19
Social > Nonsocial for neutral	1.184	<.001***	0.658	1.711
Negative > Positive for social	0.499	.129	−0.103	1.1
Negative > Neutral for social	0.496	.124	−0.096	1.088
Positive > Neutral for social	0.003	1	−0.701	0.707
Negative > Positive for nonsocial	1.5	<.001***	0.762	2.239
Negative > Neutral for nonsocial	1.26	.001***	0.47	2.05
Positive > Neutral for nonsocial	0.003	1	−0.701	0.707
P300 (320–420 ms)				
Negative > Positive	1.304	<.001***	0.701	1.908
Negative > Neutral	1.698	<.001***	1.233	2.162
Positive > Neutral	0.393	.187	−0.125	0.912
LPC (420–620 ms)				
Negative > Positive	1.176	<.001***	0.671	1.682
Negative > Neutral	1.377	<.001***	0.906	1.848
Positive > Neutral	0.201	.446	−0.146	0.548

#### ERP effects of emotional content

Analyses revealed a significant main effect of emotional content during the EPN (200–320 ms), *F*(1, 23) = 18.333, *p* < .001, η_p_^2^ = .444, the P300 (320–420 ms), *F*(1, 23) = 37.196, *p* < .001, η_p_^2^ = .618, and LPC (420–620 ms) time windows, *F*(1, 23) = 36.996, *p* < .001, η_p_^2^ = .617 (see Fig. 4b), all quantified in the posterior ROI. The respective relevant post hoc tests are summarized in Table 3.

#### Social × emotional content interactions

Averaged ERPs revealed a significant social × emotional content interaction during the P1 (80–120 ms) quantified at PO7/PO8 electrodes, *F*(1, 23) = 6.662, *p* = .003, η_p_^2^ = .225, and during the EPN (200–320 ms) quantified in the posterior ROI, *F*(1, 23) = 3.601, *p* = .035, η_p_^2^ = .135 (see Fig. 4a). The respective relevant post hoc tests are summarized in Table 3.

## Discussion

It is generally understood that social information is of high intrinsic relevance for the human species, likely having fueled the evolution of a dedicated social brain that is nowadays investigated by the still young field of social cognitive affective neuroscience (Cacioppo & Berntson, 1992; Dunbar, 1998, 2009; Hariri et al., 2002; Keltner & Kring, 1998; Lieberman, 2007; Porges, 2003; Tomasello, 2014). Accordingly, previous fMRI results suggest that social content may represent a distinct stimulus dimension (Britton et al., 2006; Frewen et al., 2010; Goossens et al., 2009; Hariri et al., 2002; Norris et al., 2004; Scharpf et al., 2010; Vrtička et al., 2011, 2013), and that the human social brain may be highly sensitive to the mere presence of social information (Tso et al., 2018). Not much is known, however, about the temporal unfolding of neural activity underlying social (vs. nonsocial) information processing, and it remains largely unresolved how social content may interact with other stimulus dimensions during stimulus processing, particularly with emotional content in terms of intrinsic pleasantness/hedonic valence. Here, we extended previous EEG data (Bayer et al., 2017; Okruszek et al., 2016) by showing that social content impacts very early stages of stimulus processing, reflected in modulation of the P1 and subsequent ERP components of short latencies. Social content therefore likely represents a unique stimulus dimension that is appraised during one of the first of a series of relevance checks (see, e.g., Sander et al., 2005; Scherer, 2009). In addition to a long-lasting main effect of emotional content, our findings furthermore demonstrated an interaction between social and emotional relevance at the level of the P1 and a subsequent ERP component, albeit with different interaction patterns across the two time windows. These interactions indicate that early stimulus relevance checks include both social and emotional stimulus characteristics, and that both sources of relevance of incoming information are integrated very rapidly during stimulus appraisal. Such pattern implies that social and emotional relevance is neurally appraised automatically and/or unconsciously, a notion further bolstered by the fact that neither emotional valence nor social content of pictorial stimuli were relevant for the task participants were performing during EEG data acquisition. In line with our expectations, the social by emotional content interaction pattern at the EPN component reproduced the interaction pattern previously described using the same stimuli in an fMRI study (Vrtička et al., 2013), and source estimations located such integrative processing of both stimulus dimensions at highly similar neural sites within the brain. The implications of our findings, also in relation to the appraisal theory of emotion, are outlined in more detail below.

### Effects of social and emotional content in ERPs

The most interesting finding of the present study is an ERP modulation by both social and emotional content—revealed by analyses excluding and including neutral stimuli—during complex visual scene processing that occurred as an increased occipitotemporal positivity (and as its counterpart—a frontocentral negativity) between 200 and 320 ms. This ERP effect was characterized by (a) a main effect of social content (social > nonsocial; for emotional as well as neutral images), (b) a main effect of emotional content (negative > positive and negative > neutral), and (c) a social by emotional content interaction. The latter interaction consisted of stronger effects of social content in positive and neutral as compared to negative images, and of an enhanced difference between valence conditions (negative > positive and negative > neutral) in nonsocial as compared with social emotional images. Not only does this pattern strongly resemble the previously observed social × emotional content interaction effect in fMRI data using the same emotional stimuli (Vrtička et al., 2013), but the estimated sources of the ERP effects from the current study show an intriguing overlap with the anatomical locations of the fMRI activations, particularly in the (right) FG and aSTG.

Within this latency, a relative negativity over occipitotemporal sites—associated with enhanced sensory encoding resulting from involuntary capture of attention to emotional content—has been reported across a wide range of experimental tasks and stimulus domains, including words, faces, and complex scenes (e.g., Bayer & Schacht, 2014; Junghoefer et al., 2001; Schacht & Sommer, 2009a; Schupp et al. 2007). Taken these findings into account, our results suggest that social content can counteract a general, and often reported (Delplanque, Lavoie, Hot, Silvert, & Sequeira, 2004; Keuper et al., 2013; Smith, Cacioppo, Larsen, & Chartrand, 2003; Zhang et al., 2014), bias for negative information at early processing stages. What is concerning the distribution of the ERP modulation to emotional and social relevance found in our study, however, we reckon that it does not resemble the typical EPN distribution but rather shows similarities to other N2-like effects previously been reported for increased attention allocation to emotional stimuli (e.g., Feng et al., 2012; Lin et al., 2015).

Interestingly, interactive processing of pictures with social and emotional content became already evident in ERPs of shorter latencies (between 80 and 120 ms), namely at the P1 component. Albeit the overall interaction pattern slightly differed from that in the subsequent time window, again the positive images benefited from increased social relevance during this stage of processing (as compared with both negative and neutral images). The P1 component is thought to reflect attention allocation during sensory processing of stimuli in the extrastriate visual cortex (i.e., being amplified for attended relative to unattended information; Di Russo, Martinez, & Hillyard, 2003; Hillyard & Anllo-Vento, 1998; Luck, Woodman, & Vogel, 2000). Our findings therefore indicate a processing advantage of particularly positive social information during stimulus encoding—even in the absence of direct goal/need relevance of emotional valence and social content in terms of participants’ task instructions. Importantly, the above effects were independent of low-level stimulus properties such as luminance, contrast, and spatial frequency, and the social content effect cannot be explained by arousal, either.

The two so far available EEG studies that directly manipulated social relevance (Bayer et al., 2017; Okruszek et al., 2016) reported an effect of social content, but neither main effects of emotional valence nor a social by emotional content interaction at the P1 component. Importantly, however, both studies only included negative and neutral stimulus materials. Interestingly, other investigations on emotion processing that also included stimuli of positive valence, demonstrated amplification of the P1 amplitudes by positive emotional content, for example, during word and face processing (Bayer, Sommer, & Schacht, 2012; Rellecke, Palazova, Sommer, & Schacht, 2011). In contrast, there are reports of early negative (vs. positive and/or neutral) emotion effects on the P1 component, again during word processing (Keuper et al., 2013; Zhang et al., 2014), but also during face processing (Smith et al., 2003), as well as when participants were viewing emotional pictures from the IAPS database (Delplanque et al., 2004). Furthermore, the P1 was previously attributed a role as a marker of the early detection of social signals (e.g., by using paradigms which contrasted the visual processing of social versus nonsocial objects; Herrmann, Ehlis, Muehlberger, & Fallgatter, 2005), or required participants to detect the presence of a human face in a complex scene (Cauchoix, Barragan-Jason, Serre, & Barbeau, 2014). To fully understand what specifically determines stimulus relevance and its influence on perceptual processing, it seems indispensable to consider other content differences within and across the emotion dimension. Among them, social aspects might convey the most important information.

Besides more general aspects of early neural social and emotional content encoding discussed above, one may ask the question why particularly social positive (vs. nonsocial positive) stimuli entailed early attention allocation during visual processing in our study. Positive social images used here included scenes that depicted parents interacting with their children, friends having a good time together, or happy moments in the context of romantic relationships. All of these images thus portrayed a social context of safety, security, and connectedness. In turn, nonsocial positive images showed animals, food, and appealing nature scenes (e.g., tropical beaches, sunsets). One possible mechanistic explanation of our ERP effects being mainly driven by the social positive images may thus be that this stimulus category contained a particular kind of relevance that drew early attention allocation. Such a notion would accord with findings from a recent study that found increased P1 amplitudes to neutral faces previously associated with monetary rewards and thus positive motivational relevance in a social context (Hammerschmidt, Sennhenn-Reulen, & Schacht, 2017). Similarly, another study (Beckes, Coan, & Morris, 2013) reported early attentional biases toward securely conditioned faces at the P1 component during an implicit face conditioning task, the latter effect likely representing an increase in the approach relevance of secure social bonds per se, without any added positive motivational relevance. Particularly the findings of the study by Beckes et al. (2013) would support the data obtained here, where positive social relevance was intrinsic to the depicted scenes as they were not previously associated with any rewarding value. We may therefore speculate that in our study, information pointing toward social safety and security, rather than nonsocial comfort, was particularly relevant for participants. A first relevance check during the appraisal of complex visual social emotional scenes could therefore represent a rapid assessment of information to fulfill a basic motivation to feel socially safe and secure, a computation that is not required when the processed information is nonsocial.

Interestingly, when also considering ERP effects for the neutral condition using the full-factorial 2 × 3 analysis, no ERP modulation to neutral images occurred at the P100 between 80 and 120 ms, but a social > nonsocial effect very similar to the effect observed for positive images was present at a slightly later time window between 200 and 320 ms during the EPN. This pattern implies that while initially, particularly social positive information may be processed more differentially according to its social versus nonsocial content, such effect extends to neutral social versus nonsocial content appraisal during a subsequent processing stage. An according sequential effect in the processing of social versus nonsocial information as a function of its positive versus neutral valence may be attributable to a previously observed effect of preferential early attention allocation to positive (vs. neutral) stimuli, for example during word and face processing (Bayer et al., 2012; Rellecke et al., 2011). What remains intriguing, however, is the fact that during the P1, ERP responses only differed for positive and not negative (and neutral) pictures in the present study, although early P1 effects to negative (vs. positive and/or neutral) stimuli including words (Keuper et al., 2013; Zhang et al., 2014), faces (Smith et al., 2003), as well as IAPS pictures (Delplanque et al., 2004) have been reported before. Future investigations are therefore needed to replicate and further characterize this early social by emotional content processing pattern by also taking into account the context within which information is processed, and ideally also probing for interindividual differences that may shed more light on the source of the observed attentional biases.

Based on previous literature, we were expecting modulation of ERPs also during later stages of affective picture processing, namely during the P3/LPC time windows (e.g., Bayer & Schacht, 2014; Cuthbert et al., 2000; Schupp et al., 2007; for a review, see Olofsson et al., 2008), in particular in response to negative pictures. Indeed, a long-lasting main effect of emotional content occurred in the present study, persisting for several hundred milliseconds, with increased ERP amplitudes to negative compared with positive and neutral images. However, this effect, although slightly changing in topography over time, did not resemble centroparietal positivities typical for P3/LPC effects, but rather consisted of increased bilateral positivities over occipital electrode sites and—as their counterparts—frontocentral negativities. It appears difficult to compare our present data with previous studies that refrained from depicting topographical maps on the effects of interest. In a previous study that employed the same task as we used in our study (decisions on intact pictures vs. their scrambled versions), grand averaged ERPs to more arousing pictures showed high similarities to our findings (Rosenkrants et al., 2008). The specific, and presumably, task-related conditions under which typical emotion-related ERPs, like the EPN and subsequent P3/LPC components are elicited, needs further investigation. Furthermore, it is noteworthy that we only observed a main effect of social content and a social × emotional content interaction during the P1 and/or the EPN. In other words, despite a persisting emotional content effect in later ERP components, the social content effect appeared to “fade out” after about 420 ms. Within this context, the component process theory of emotion we refer to here (see also below) postulates that the different appraisal checks can be—and likely are—processed in parallel, but also that “the result of a prior processing step (or check) must be in before the consecutive step (or check) can produce a conclusive result with efferent consequences” (Sander et al., 2005, p. 322). Our data therefore tentatively suggest that the processing of social content may be concluded somewhat earlier than the appraisal of emotional content, the latter continuing to show in later ERP components. Whether this is due to differences in processing speed and/or the involvement of fewer and/or different appraisal processes needs to be established by follow-up experiments.

### Theoretical implications

According to the component model of emotion processing (see, e.g., Sander et al., 2005; Scherer, 2009) situated within the larger realm of appraisal theories of emotion, stimulus appraisal consists of a series of appraisal checks, of which the first one is devoted to relevance detection in terms of a “selective filter through which a stimulus or event needs to pass to merit further processing” (Scherer, 2009, p. 3463). Within this framework, relevance detection is argued to comprise information evaluation in terms of novelty (i.e., suddenness, familiarity, and/or predictability), intrinsic pleasantness (i.e., negative vs positive [vs. neutral] valence), and goal/need relevance (i.e., whether the assessed information accords to or obstructs the current goals and needs of the organism). First evidence on the neural temporal sequence of appraisal processes and in particular relevance detection in terms of novelty and pleasantness is already available (van Peer et al., 2014), pointing to a sequence of novelty (between 200 and 300 ms) to intrinsic pleasantness (between 300 and 400 ms), and finally a novelty × intrinsic pleasantness interaction (between 700 and 800 ms). However, such sequence originates from a particular task comprising a few novel items amongst many repeated distractors (i.e., oddball paradigm) not dissociating between stimulus dimensions in terms of social and emotional content. Using a different experimental paradigm and by directly manipulating social and emotional stimulus content, we show here that relevance detection may already occur as early as 80 to 120 ms after stimulus onset at the P1 and continue during the EPN (between 200 and 320 ms) component, and that such relevance detection could be characterized by an interactive processing of social and emotional information. Our new findings therefore tentatively suggest that, apart from novelty, intrinsic pleasantness, and goal/need relevance, social content evaluation may represent an additional, independent early relevance check. At the same time, our data imply that different relevance checks may occur simultaneously, even already as early as 100 ms after stimulus onset, and that the outcomes of these independent relevance checks are integrated from the very beginning of stimulus appraisal.

In terms of relevance detection being suggested to act as a first “selective filter through which a stimulus or event needs to pass to merit further processing” (Scherer, 2009, p. 3463), our new data could imply that early (visual) attention allocation is not only influenced by the emotional valence (negative vs. positive vs. neutral) of incoming information, but also its social versus nonsocial content. Such early integrative processing of several stimulus dimensions appears to make a lot of sense, particularly regarding social and emotional content of information, because social versus nonsocial cues of different valence may signal distinct situational properties that require disparate psychological and physiological responses. On the one hand, particularly negative information—regardless of its social or nonsocial content—is assumed to be highly relevant to the human organism because it represents a potential threat to the bodily integrity as well as psychological well-being. Accordingly, the rapid appraisal of negative information appears crucial for triggering defensive behavior, associated psychophysiological responses, and emotional feelings as a means of protection. On the other hand, because positive and neutral information should in general not hold such immediate biological salience in terms of survival, early attention allocation could be expected to be somewhat less pronounced. However, it has been proposed that for humans, social information may hold an intrinsically higher relevance due to its direct link to guiding physiological responses and behavior (Hariri et al., 2002; Keltner & Kring, 1998), and that the human social brain may be highly sensitive to the mere presence of social information (Tso et al., 2018). It therefore appears plausible that an additional early appraisal check may exist, devoted to determining whether the attended information is social or not, and that such appraisal check may particularly enhance the processing of social positive and neutral information. As evident from our full-factorial social (2) × emotional (3) content interaction during the EPN time window, there indeed was such pattern evident from the averaged ERP responses in terms of a significant social > nonsocial activation difference for both positive and neutral images.

In future studies, it will be important to replicate and extend the present findings by investigating even more stimulus dimensions within one experimental design to capture relevance detection to novelty, intrinsic pleasantness, goal/need relevance, and social content evaluation, and particularly their interrelations, by using different experimental paradigms in different contexts, ideally also including interindividual differences to assess possible underlying motivational states.

### Limitations

One potential limitation of the present study is the inclusion of female participants only. It is possible that females appraise complex visual scenes differently from males in terms of particular combinations of social and emotional stimulus content possibly being attributed with a different relevance as a function of participant sex. For example, one previous EEG investigation reports sex differences in P1 amplitude modulation during emotional face viewing overall and particularly when faces were emotionally positive/rewarding (Pfabigan, Lamplmayr-Kragl, Pintzinger, Sailer, & Tran, 2014). At the same time, there is evidence for a female negativity bias at the N1 and N2 amplitudes during passive viewing of emotional images (Gardener, Carr, MacGregor, & Felmingham, 2013; Lithari et al., 2010). Future studies are needed to resolve this issue by including both female and male participants and directly comparing brain responses between the two sexes.

Another possible limitation in the context of appraisal theories of emotion is the fact that we only assessed the two stimulus dimensions of social and emotional content, the latter representing the intrinsic pleasantness relevance check, but not other relevance checks as part of the component model of emotion processing (see above). More research is therefore clearly needed to obtain a more comprehensive image of the temporal unfolding of stimulus appraisal and relevance detection on a neural level.

Finally, while the stimuli used here did not differ in valence and arousal ratings across social versus nonsocial categories, there was a significant difference in arousal ratings between negative > positive > neutral images. Although this is a rather common finding, it somewhat limits the interpretation of the reported emotional content effects in terms of valence or intrinsic pleasantness appraisal, because intrinsic differences in arousal may also point to other possible mechanisms more likely related to stimulus intensity.
